# Identification and interpretation of gait analysis features and foot conditions by explainable AI

**DOI:** 10.1038/s41598-024-56656-4

**Published:** 2024-03-12

**Authors:** Mustafa Erkam Özateş, Alper Yaman, Firooz Salami, Sarah Campos, Sebastian I. Wolf, Urs Schneider

**Affiliations:** 1 Fraunhofer IPA, Nobelstrasse 12, Stuttgart, Germany; 2 Clinic for Orthopedics, Heidelberg University Hospital, Schlierbacher Landstrasse 200a, 69118 Heidelberg, Germany

**Keywords:** Biomedical engineering, Computer science, Scientific data, Computational science

## Abstract

Clinical gait analysis is a crucial step for identifying foot disorders and planning surgery. Automating this process is essential for efficiently assessing the substantial amount of gait data. In this study, we explored the potential of state-of-the-art machine learning (ML) and explainable artificial intelligence (XAI) algorithms to automate all various steps involved in gait analysis for six specific foot conditions. To address the complexity of gait data, we manually created new features, followed by recursive feature elimination using Support Vector Machines (SVM) and Random Forests (RF) to eliminate low-variance features. SVM, RF, K-nearest Neighbor (KNN), and Logistic Regression (LREGR) were compared for classification, with a Majority Voting (MV) model combining trained models. KNN and MV achieved mean balanced accuracy, recall, precision, and F1 score of 0.87. All models were interpreted using Local Interpretable Model-agnostic Explanation (LIME) method and the five most relevant features were identified for each foot condition. High success scores indicate a strong relationship between selected features and foot conditions, potentially indicating clinical relevance. The proposed ML pipeline, adaptable for other foot conditions, showcases its potential in aiding experts in foot condition identification and planning surgeries.

## Introduction

Gait analysis relies on torque and angle profiles of the focused joints that are obtained by averaging across multiple strides, which as a result, provides mean and standard deviation values along the whole gait cycle^1^. However, evaluation of gait profiles is difficult and time-consuming due to the need for manual examination by medical experts and physicians. These professionals rely heavily on their experience, expertise, and familiarity with patient demographics. Despite the data being quantitative, their evaluations (e.g. the intensity of the foot deformity) may inherently contain a degree of human bias. Keep in mind that, experts and physicians not only check the gait data but also use different assistive tools (e.g. foot images) and do physical examinations to eliminate errors in the identification and intensity of the foot deformity. Computer-based tools aid in managing large datasets and can simplify data by computing major features, such as minimum or maximum specific joint angles. Additionally, automatic feature generation could be implemented using mathematical tools, e.g. principal component analysis (PCA)^2^ or linear discriminant analysis (LDA). However, the resultant features may not correspond to the kinematic characteristics of the joints, and hence may not hold clinical relevance.

From a clinical standpoint, foot conditions significantly impact an individual's mobility, quality of life, and risk of injury. Early diagnosis and intervention can greatly enhance treatment outcomes, reduce healthcare costs, and improve patient well-being. Therefore, the capacity to identify foot conditions either through the entire gait data (i.e., time series normalized to a gait cycle) or generated features is of immense clinical importance.

Moreover, the potential to automate this process through state-of-the-art Machine Learning (ML) based classification methods could expedite gait data assessment, thereby helping medical experts to provide faster and more accurate diagnoses^3^. Artificial Intelligence (AI), specifically ML, has shown exceptional promise in various fields, particularly in automating tasks and enhancing predictions. In the medical sphere, ML has contributed to more efficient diagnoses and treatment plans, and in certain areas, even surpassed human experts in diagnostic accuracy^4,5^.

However, the 'black-box' nature of ML methods often makes the interpretation of their outcomes challenging^6^. This opacity can hinder their applicability in medical fields, where understanding the rationale behind predictions is crucial for subsequent diagnoses and treatments. Consequently, Explainable Artificial Intelligence (XAI) methods, such as Shapley Additive Explanations (SHAP)^7^ and Local Interpretable Model-agnostic Explanation (LIME)^8^, have gained traction for their ability to decipher ML model predictions.

### Related work

The gait data is obtained commonly in gait labs where the motion capture cameras and sensors are kept well-calibrated and the environmental conditions are standardized. In this study, the gait data that was averaged across multiple strides and normalized to the gait cycle was used. Note that, there are numerous studies in the literature that employ wearable sensor data and often employ ML-based time-series classification to enhance the accuracy and robustness of gait analysis^1,3^.

A gait analysis method employing inertial measurement units (IMUs) attached to the shanks was proposed for estimating foot trajectory and temporal gait parameters, including stance and swing times^9^. Additionally, a gait recognition algorithm was introduced that utilized a fusion network of long short-term memory (LSTM) and convolutional neural network (CNN) to identify abnormal gait patterns by feeding preprocessed IMU data into the LSTM-CNN network for feature extraction and classification^10^. However, having a robust and reliable calculation using IMUs can be challenging since the IMU data has notable noise and drift in its linear acceleration and angular velocity data, respectively. Many filters (e.g., complementary filters) and ML algorithms were used to eliminate this noise and drift in the calculation of the rotational angles^11–14^. In addition to the robustness and reliability issues of IMUs, the attachment of these sensors to human limbs introduces an additional source of error in joint angle calculations. This error stems from the continuous deviation of their relative pose with respect to the joint axis due to the movement of the surrounding soft tissues during walking. Furthermore, their location and the orientation relative to the real joint axes may have influence on the joint angle calculations. In some studies, this issue was resolved by finding joint axis and transform the calculated angles to the coordinate system defined by the joint axis^15,16^. In some studies, other types of data, e.g., 3D point clouds generated from the camera images were fed into adversarial auto-encoders for the estimation of gait quality index^17^. Their approach has a potential to be adjusted to classify foot conditions. However, interpretability of this model could be more complex considering that it does not use input features that are easy to understand by the clinicians. It is noteworthy that the aforementioned LSTM-CNN and adversarial auto-encoders need large data, which makes them difficult to use with the gait profiles as the data.

In the literature, gait analysis has been extensively conducted using gait profiles because they offer data that is easier for non-engineers to understand. Doederlein et al. developed graphical decision matrices of foot deformities to assist physicians in disease discrimination for their diagnostic decision processes^18–22^. Bajpai et al. focused on a probabilistic approach to generate an automated gait assessment score (A-GAS) for children with cerebral palsy^23^. In a subsequent study, they used the same data and developed a neural network-based tool for identifying gait abnormalities in children with cerebral palsy (CP)^24^. The gait data can be reduced by manually extracting features: Wolf et al. presented an automated feature assessment workflow specifically for gait analysis of CP patients^25^. This approach consists of deriving time series from the original time series, computing scalar features from the derived and original time series, and evaluating computed scalar features^26^. ML methods were successfully employed in clinical gait analysis^27,28^ for the classification of CP^29^, osteoarthritis^30^, multiple sclerosis^31^, etc. In recent studies, XAI methods were employed, e.g., Layer-Wise Relevance Propagation (LRP) was used to interpret the prediction of the individual gait pattern classification model^32^. Slijepcevic et. al. employed LRP to explain the outputs of CNN, SVM, and Multi-Layer Perceptron-based classifiers in classifying lower body gait disorders. 3D ground reaction forces were employed to determine whether a person has a pathological gait pattern^33^. In their recent study, they compared the performances of CNNs, self-normalizing neural networks, random forests, and decision trees in the classification of CP gait patterns in children, and used GradCAM to explain the model outputs. It was shown that traditional ML methods achieve better results and focus more on clinically relevant regions compared to deep neural networks^34^.

In all these studies, only a few foot conditions were aimed to be identified. In this study, we propose an approach that harnesses ML for automated feature selection and foot condition identification, and XAI for prediction interpretation. This approach aims to support medical experts and physicians in their assessments, thereby improving patient outcomes. By leveraging state-of-the-art feature elimination and ML algorithms, we aim to enhance the diagnosis of various foot conditions. Moreover, we also strive to elucidate the outcomes of these ML models using XAI methods. We believe that the proposed ML pipeline, which automates feature selection, foot condition identification, and interpretation through XAI, represents a significant step forward in the fusion of technology and medicine, ultimately benefiting both medical professionals and patients alike. To the best of our knowledge, this study is the first to combine feature selection, ML classification, and XAI to use gait data and identify foot conditions.

## Methods

### Subjects

The anonymized retrospective gait data of 248 patients with 6 different foot conditions (Table 1) and 100 subjects with the typically developed feet has been collected in the course of patient care over the last two decades in the Department of Orthopedics and Traumatology, Heidelberg University, Heidelberg, Germany.

**Table 1:** Foot conditions, number of subjects, age, height, and weight.

Foot conditions (classes)	Number of subjects	Age (mean ± std)	Height(cm) (mean ± std)	Weight (kg) (mean ± std)
Tibiotalar osteoarthritis + partial ankle replacement	58	57.9 ± 11.7	170.5 ± 8.6	82.6 ± 17.7
Planovalgus	64	31.3 ± 15.6	171.9 ± 12.4	71.7 ± 20.1
Consolidated calcaneal fracture	20	52.5 ± 10.4	178.0 ± 8.2	86.6 ± 11.7
Hallux rigidus	40	58.6 ± 8.2	168.2 ± 9.6	74.8 ± 13.3
Clubfoot	41	11.8 ± 9.3	140.1 ± 28.2	41.5 ± 21.8
Cavovarus	25	19.5 ± 14.9	161.0 ± 18.9	59.7 ± 23.1
Typical feet	100	24.0 ± 15.2	159.8 ± 23.7	55.6 ± 23.9
Total Mean	348	36.5 ± 18.1	164.2 ± 11.4	67.5 ± 14.8

The demographics and the foot conditions focused in this study are listed in Table 1. The number of subjects among foot conditions (i.e. classes) varies significantly (Table 1), indicating the imbalance in data. The foot conditions are described as follows. Tibiotalar osteoarthritis involves a gradual breakdown of cartilage and inflammation in the ankle, leading to pain and mobility issues^35^. Relief and joint damage management in this condition can be achieved through partial ankle replacement. Planovalgus, characterized by a collapsed arch and inward heel tilt, causes instability during walking or standing^36^. A consolidated calcaneal fracture refers to a healed break in the heel bone, where the fractured parts have reunified to form a solid structure. Hallux rigidus induces stiffness and limited movement in the big toe, resulting in pain and difficulties while walking^37^. Clubfoot, a congenital condition, involves the inward twisting of the foot and necessitates prompt treatment for proper alignment^38^. Cavovarus, characterized by a high arch and inward heel tilt, leads to instability and difficulties in walking^39^.

Foot conditions exhibit varied age distributions. For instance, conditions such as tibiotalar osteoarthritis and partial ankle replacement tend to manifest in later life, while others like clubfoot are congenital and treated during infancy or early childhood. The height and weight distributions align with the age distributions. While these demographic variations are noticeable, they might not have significant clinical implications in the diagnosis of foot conditions. Nonetheless, the differences in age distributions are potentially relevant, given the distinct foot kinematics between children and adults during walking^40^. This differentiation is likely to play a role in the performance of the ML models.

### Retrospective data

The Heidelberg Foot Measurement Method (HFMM)^25^ was employed for data collection using a 12-camera VICON motion capturing system (Vicon Motion Systems Ltd. Oxfordshire UK). The gait data collection procedure involved a self-selected speed walking on a seven-meter walkway, and five to seven strides with good data quality were taken into account in each single trial. Vertical ground reaction force was also collected using force plates (Kistler Instruments, Winterthur, Switzerland) to segment gait cycles with respect to foot-off timing points.

The dataset contains 12 functional angles: Tibiotalar flexion, Medial arch inclination, Medial arch angle, Lateral arch angle, Subtalar inversion, Forefoot/ankle supination, Forefoot/midfoot supination, Forefoot/hindfoot abduction, Forefoot/ankle abduction, Inter MT I-V angle, Hallux adduction, Hallux flexion. These are described by Simon et. al.^25^ as follows and comprehensive illustrations are available in their published work. Tibiotalar flexion involves the rotational movement between the tibia and talus, indicated by the movement of the calcaneus and navicular, around the malleolar line, primarily in the sagittal plane. Medial arch inclination is the angle formed between the malleolar line and the line perpendicular to the plane of the medial arch, mostly within the frontal plane. Medial arch angle is the 3D absolute angle formed between the line from the medial calcaneus marker to the navicular and the first metatarsal (MT I), mostly within the sagittal plane. Lateral arch angle is the angle between the line from the lateral calcaneus marker to the fifth metatarsal head (MT V head) and MT V, primarily in the sagittal plane. Subtalar inversion is the rotation of the calcaneus around the subtalar axis, primarily within the frontal plane. Forefoot/ankle supination refers to the angle between the metatarsal head line and the malleolar line, mostly within the frontal plane. Forefoot/midfoot supination is the angle between the metatarsal head line and the metatarsal base line, mostly within the frontal plane. Forefoot/hindfoot abduction is the rotation of the metatarsal head line relative to the calcaneus, primarily in the transverse plane. Forefoot/ankle abduction is the rotation of the metatarsal head line relative to the malleoli line, mostly in the transverse plane. Inter MT I–V angle is the absolute angle between the first metatarsal (MT I) and the fifth metatarsal (MT V) when projected into the transverse plane. Hallux adduction is the angle between the first metatarsal (MT I) and the hallux projected into the plane defined by the metatarsal heads and the foot axis, primarily in the transverse plane. Hallux flexion is the rotation between the hallux and the first metatarsal (MT I) around an axis perpendicular to the foot axis, in the plane of the foot axis and metatarsal head line, mostly within the sagittal plane.

Each functional angle data was split into strides using foot-off timing points, averaged point-wise across 5–7 strides from one single trial, and normalized to a percentage gait cycle (i.e. between 0 and 100%) to obtain the mean time series in the retrospective dataset. In addition, the standard deviation (std) time series were calculated to provide point-wise variation among strides. Each time series consisted of 101 data points representing the gait cycle from 0 to 100%. Note that, only the data of the affected legs were selected from the dataset.

### Feature engineering

**Figure 1:**
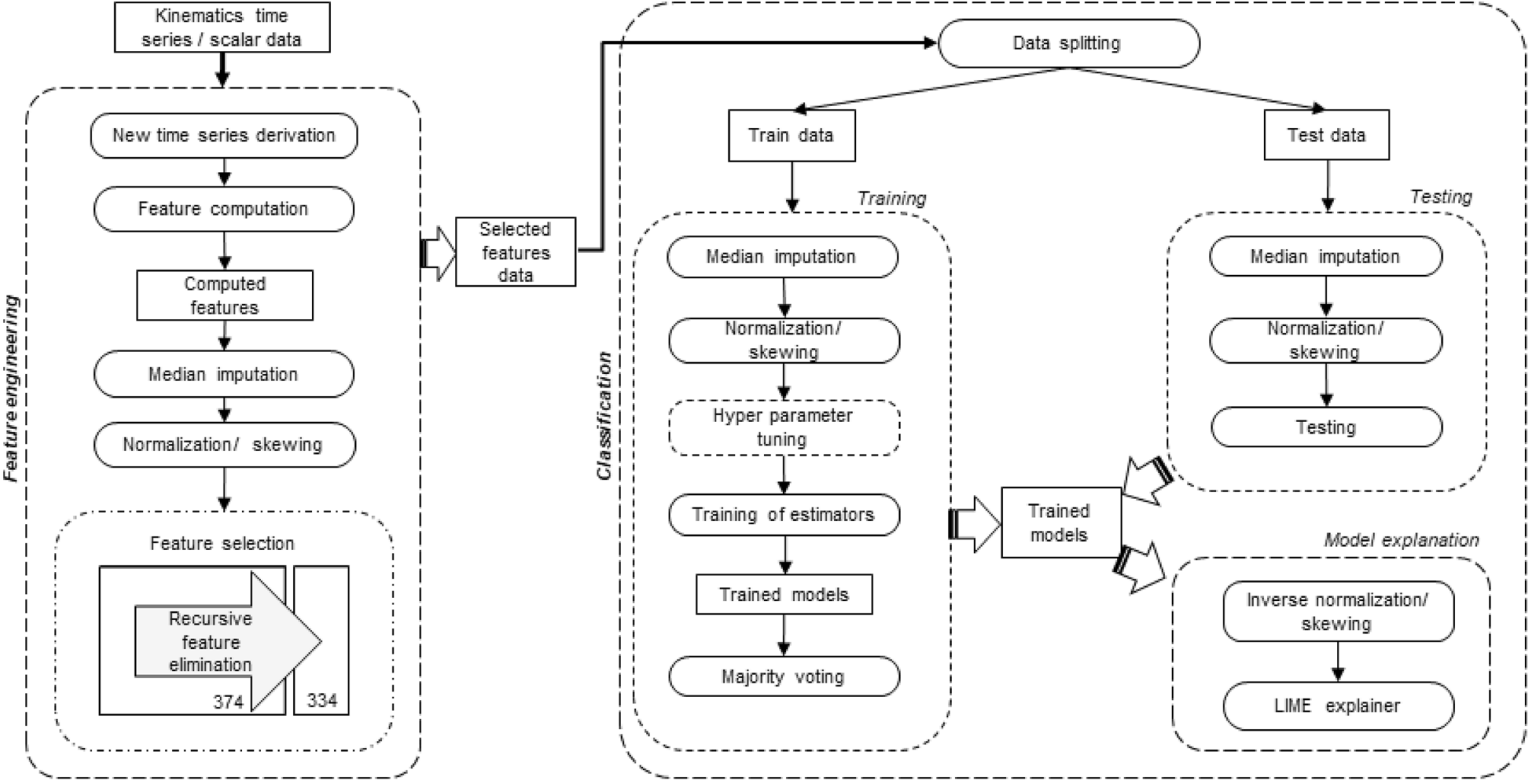
Overall method flowchart.

The feature extraction and selection steps are denoted in Fig. 1. Two new time series were derived and scalar features were computed from all of the time series as in the study of Wolf et. al.^26^. Subsequently, median imputation was performed to fill the missing values as preserving the data distribution. The data was normalized to rescale the values between 0 and 1.This was done to prevent over- or under-representation of time series with extremely high or low magnitudes compared to the other time series. Subsequently, features with low variance were eliminated by ML-based feature selection algorithms since they might have negligible effect in classification. In the subsections below, these steps are explained in detail.

#### New Time series derivation

In order to extract further information from the mean time series, two new time series (i.e. the first gradient and difference from normative) were derived similarly to the automated feature assessment workflow of Wolf et al.^26^. The first gradient time series illustrates the changes occurring throughout the gait cycle, while the latter indicates how the time series deviates from the average typical foot (i.e., normative). The first gradient “$V$” of the mean time series “$U$” was calculated in a discrete domain according to the Formula (1):

1 $$V\left[k\right]= \frac{1}{2}(U\left[k+1\right]-U\left[k-1\right])$$

where “k” is the data point index within a gait cycle, which is in our case a number between 0 and 100. The difference from the normative time series “$D$” was calculated according to the Formula (2):

2 $$DN\left[k\right]= \left|U\left[k\right]-{U}_{norm}\left[k\right]\right|$$

For each functional angle, reference normal time series ${U}_{norm}$ was calculated by averaging the corresponding time series across all subjects with typical feet.

#### Feature computation

Each time series was segmented to gait phases (stance and swing) since segmenting enhances the physiological relevance of the peak values and other computed features for each gait phase, as suggested by Wolf et al.^17^. Computing scalar features from the segmented time series significantly decreases the ML input size causing lesser but still representative data while reducing the complexity of the model. The features explained below were separately computed for the stance and swing phases of the gait cycle, except the range of motion feature, which was calculated for the whole gait cycle.

**Figure 2:**
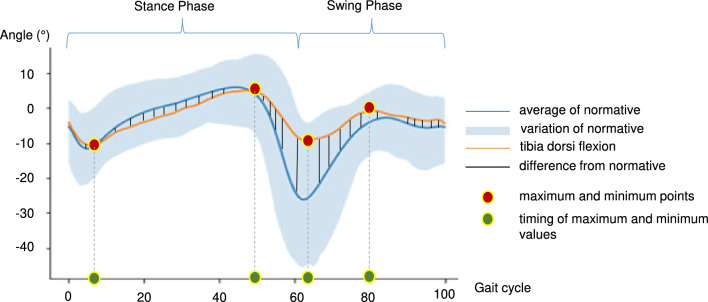
Sample features computed from an averaged time series normalized to a gait cycle. The orange line shows the tibiotalar flexion of the left foot for a subject with hallux rigidus. The blue line and band show the mean value and variation of the reference typical feet data. Red and green circles show the maximum and minimum values on the data (orange line) and their corresponding timing values on the x-axis, respectively. “Difference from normative” is calculated as a feature by averaging the differences denoted by the vertical black lines.

For mean and derived time series, the computed scalar features were as follows: Minimum and maximum values and their gait cycle instances, i.e., the temporal position in the gait cycle, referred to as "timing" (x-axis in Fig. 2) for simplicity throughout the rest of the article. For the standard deviation (std) time series, only the maximum value and its corresponding timings were taken into account as features. This decision was based on the rationale that certain foot conditions might induce more pronounced variations in gait patterns across strides and these features will supply this information. As indicated in Table 2, each functional angle was associated with 15 features separately for the stance and swing phases. The total number of features increased to 31 when computed separately for these phases (15 × 2 = 30), with the addition of the range of motion as an extra feature. The total number of extracted features for all functional angles can be determined by multiplying the number of functional angles by the number of features, resulting in 12 × 31 = 372. Furthermore, the dataset incorporated the "Foot_off" scalar value, denoting the time when the foot is off, to distinguish between the stance and swing phases. Two "Foot_off" values were included, one for the mean and one for the standard deviation of it. Consequently, the overall number of features for each subject reached 374.

**Table 2:** Computed features for the time series.

Time series	Max value	Timing max	Min value	Timing min	Average difference from normative
Mean	X	X	X	X	
Std	X	X			
First gradient	X	X	X	X	
Difference from normative	X	X	X	X	X

Figure 2 shows the computed features on an example mean time series “Tibiotalar Flexion of Left Foot for a Hallux Rigidus Subject” and the difference from the normative.

#### Median imputation and normalization

We employed median imputation to the time series data to fill missing values while preserving the data distribution. Similar to the study on missing longitudinal data^41^, the missing values were filled with the median of the values in the corresponding timing of the other subjects having the same foot condition. The input values were of different scales and had to be normalized between 0 and 1 for simplifying the learning process of the ML algorithms^42^. Moreover, a natural logarithmic transformation was applied to skew the input distribution to the normal distribution. However, no significant improvement through skewing was observed.

#### Feature selection

Computed scalar features ended up with 374 scalar values per subject. Not just for computational applicability, but also for problem-related specification^43^, the most useful subset of features was selected. Recursive feature elimination with cross-validation algorithm of Scikit-Learn module^44^ was used for feature selection. This algorithm was implemented with a Support Vector Machine (SVM)^45^ algorithm as an estimator, which was trained recurrently with the newest subset of the features, the least important of which (regarding model coefficients) was then eliminated for the next trial. The feature subset securing the maximum accuracy in the estimator algorithm was then assumed to be the ideal subset. In addition to this algorithm, a Random Forest (RF)^46^ model was trained with all computed features and the importance parameter^44^ was used to select the most relevant features by eliminating the ones below a threshold. The intersection of the selected relevant feature subsets of these two algorithms was used as the optimum subset for training the classifier models. For this optimum subset, 334 of the 374 features were selected, which was the intersection of the outcomes of the aforementioned algorithms.

### Classification for the identification of foot conditions

Figure 1 shows the classification steps. Briefly, the dataset was split for training and testing and median imputation and normalization were applied to each subset, separately. In training, hyperparameters of the ML models were optimized and then models were trained. In testing, the trained models were tested with the split unseen test data.

#### Dataset splitting

The dataset consists of the selected scalar features of the functional angles (as inputs) and their foot conditions (as outputs). The dataset was split into training and testing groups with a ratio of 0.85 to 0.15 in a stratified way, respectively. The test data was isolated to guarantee that there was no information leakage between the train and test datasets. Yet, the abovementioned preprocesses (median imputation and normalization) was executed for the training and test data separately. Note that the test data was scaled using the scaling factors provided in the scaling of the training data to avoid dimension mismatches and scale impropriety.

#### Machine learning algorithms

For the classification of the aforementioned foot conditions (see Table 1), 5 conventional ML algorithms were evaluated in multiclass classification strategy and their results were compared: Support Vector Machines (SVM)^45^, Random Forest (RF)^46^, Logistic Regression (LREGR)^47^, K-nearest Neighbor (KNN)^48^, and a majority voting (MV)^44^ algorithm were trained separately. The MV model was implemented with a weighted soft voting technique that uses the trained SVM, KNN, RF, and LREGR models and gives the weighted voting outcome of them as the final output.

#### Hyperparameter tuning

Within the cross-validation, SVM, KNN, and RF models were tuned with a randomized search algorithm before training for finding the best hyperparameters^49^. For the LREGR model, a computationally expensive grid search algorithm was required for ensuring model convergence. The hyperparameters of SVM, RF, LREGR, and KNN are as below:

- SVM: Type of kernel, kernel coefficient (gamma), regularization parameter
- RF: Number of trees in the forest, split quality criterion, minimum required samples for splitting a node, minimum required samples for being a leaf node, maximum depth of trees, maximum number of features for splitting, existence of bootstrapping
- LREGR: Regularization strength, type of solver algorithm, maximum number of iterations
- KNN: Type of distance metric, type of weight function, type of algorithm for computing nearest neighbor, size of leaf (for the requiring algorithms)

MV weights were optimized through a randomized search algorithm after training the first four models (SVM, KNN, RF, and LREGR). Further details about the mentioned hyperparameters of the models can be found in the documentation of Sklearn Framework for^44^.

#### Training and testing

The tuned models were trained without a hard limit on the number of iterations. Instead, a stopping criterion was set with a threshold of change in the loss by the value of 1e-4 for two consecutive iterations. 3-times repeated leave one out cross-validation was applied, similar to the approach used by Bajpai et. al.^50^. While the first 4 models do not need a special order of training, the MV model has to be trained at last, since it uses the outputs of the first 4 models as inputs.

To quantify the classification success of the models, multiclass averages of balanced accuracy, recall, precision, and F1 scores of the models were calculated using the counts of true positives (TP), true negatives (TN), false negatives (FN), and false positives (FP). Table 3 lists the formulas of the measures and their descriptions^51^. The highness of these performance measures altogether, without them being correlated, indicates the success of the classification^52^. These scores were calculated for the isolated test dataset.

**Table 3:** Performance measures, their formulas, and definitions (31).

Measure	Formula	Description
Balanced accuracy	$\frac{1}{2}* \left(\frac{TP}{TP+FN}+\frac{TN}{TN+FP}\right)$	Average per foot condition effectiveness of a classifier model, calculated for each foot condition by weighting the prevalence of it
Recall	$\frac{TP}{TP+FN}$	Effectiveness of a classifier model to identify foot conditions if calculated from sums of per-subject decisions
Precision	$\frac{TP}{TP+FP}$	An average per-foot condition agreement of the real foot conditions of subjects with those predicted by the classifier model
F1 score	$2*\frac{Precision*Recall}{Precision+Recall}$	Relations between positive labels of data and those given by the classifier model, based on sums of per-foot condition decisions

#### Model interpretation

Before model interpretation, the features were rescaled to reach their original values. The input features of the trained models were evaluated in terms of their relation to the decisions of the models. For this purpose, LIME algorithm was used, which observes the effect of each input feature on the output by doing perturbations on the inputs^8^.

The LIME algorithm assigns weights and intervals to each feature, indicating the extent of influence each feature has on classification output of the model and the interval within which the feature exerts the greatest influence. However, since the intervals of the feature values are more complex to be interpreted by the clinicians, we only considered the weight of the features. We have calculated the mostly weighted 5 features in average for a randomly selected 20 subjects to be classified in each foot condition, for which these 5 features are then defined as the most relevant in classifying them. As from the nature of the LIME algorithm in our case, the magnitude of the weight was decreasing significantly after the first 5 features, which allowed us to be able to interpret the models using them.

### Ethics approval and consent to participate

Ethics approval was obtained from Ethical Commission of the Medical Faculty of the University of Heidelberg “Ethikkommission der Med. Fakultät der Universität Heidelberg“ S-850/2019. All methods and procedures were carried out in accordance with relevant guidelines and regulations. Contacting patients and asking for their consent for a retrospective analysis of their data for the described research purpose is not done in this study. The number of persons to be contacted would be large, and in some cases the contact would be in relation to medical care that took place several years ago, which means that a corresponding change of address of the persons concerned is not unlikely. The protection of the anonymized biomechanical data is of secondary importance compared to the described research interest, because in particular.

- the data to be evaluated are already available at the research centre and the original collection took place in the context of routine medical care and the data are to be processed here only for research purposes, and
- for the purpose of the research, only persons who were already authorized to inspect personal data on the occasion of routine medical care are allowed to do so, and
- Personal data will not be passed on to external bodies.
- A waiver for the need of informed consent was granted by the Ethical Commission of the Medical Faculty of the University of Heidelberg “Ethikkommission der Med. Fakultät der Universität Heidelberg“.

## Results

### The tuned hyperparameters

The tuned hyperparameters of the aforementioned models are as follows. In the SVM a linear kernel with a regularization parameter (C) of 70, was used. In the RF, the number of trees in the forest was selected as 6984, the GINI impurity criterion was used, the minimum required subjects for splitting a node was selected as 2, and the minimum required subjects for being a leaf node was selected as 1, max depth of trees was limited to 40, the square root of the total feature number was selected as the maximum feature number to be considered and a bootstrapping was implemented. In the LREGR, the inverse of regularization strength (C) was selected as 0.7 and the solver was selected as stochastic average gradient (SAG) algorithm. The LREGR was executed with a maximum iteration number of 5000 for converging the model. In the KNN, the Manhattan distance metric was used for 6 neighbors with uniformed weights and a KDTree algorithm was used for nearest neighbor calculation, for which a leaf size of 10 was used.

### Training and classification scores

**Figure 3:**
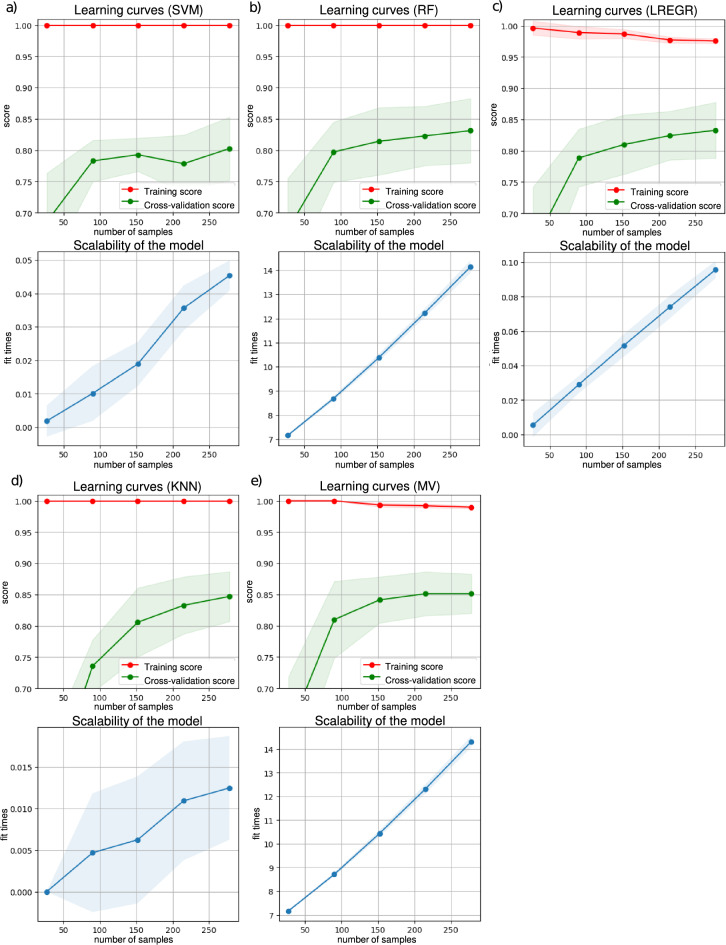
Training graphs of the models. The learning curves and scalability of the model graphs for (**a**) SVM, (**b**) RF, (**c**) LREGR, (**d**) KNN, (**e**) MV. X-axis denotes the included number of samples while y-axis refers to the average accuracy (score) and model training times (fit times) in the learning curves and scalability of the model graphs, respectively.

The learning curves and scalability graphs are shown in Fig. 3 for SVM, RF, LREGR, KNN, and MV respectively. The faded areas show the standard deviations in each. The learning curve graphs show the average accuracy score for training and cross-validation sets. In all graphs, the x-axis corresponds to number of samples, not the number of iterations. When there is sufficient number of samples, training gave the score of 1.0, showing that the input features provided to the classifier models are in general distinctive. Training scores were descended with the increasing number of samples, which suggests that the model is becoming better in generalization with the increasing number of samples. Successful cross-validation scores are attained only when a sufficient number of samples are included. The cross-validation score graphs for models that do not entirely converge suggest that additional data could lead to more successful model training. The gap between the training and cross-validation curves indicates a little yet natural overfitting that may decrease as the data size becomes bigger with the new data. In the scalability graphs, the linear trends in fit times (model training times) along with the change in the number of samples indicate the robustness of the models.

The average test scores for each model are shown in Table 4 below. All of the trained models achieved high scores in every metric, meaning that not only the foot conditions were predicted correctly, but also false prediction was too rare.

**Table 4:** Test scores for each model.

Trained model	Mean balanced accuracy	Mean recall	Mean precision	Mean F1 score
SVM	0.83	0.83	0.83	0.82
LREGR	0.83	0.83	0.83	0.83
RF	0.84	0.84	0.84	0.84
**KNN**	**0.87**	**0.87**	**0.87**	**0.87**
**MV**	**0.87**	**0.87**	**0.87**	**0.87**

Peak scores are in bold.

### Model interpretation outcomes

In Table 5, the most relevant kinematic features obtained using LIME algorithm are listed for each foot condition. In each feature name in Table 5, the following information is to be read: The short name of the functional angle the feature belongs to (as in Table 1), the type of the derived time series if not itself, the segment of the gait cycle to which the feature belongs to. If the standard deviation time series is used instead of the mean time series, it is indicated with a |STD| in the short time series name. The length of the bars corresponds to the number of selections of a feature as one of the most dominant ones. By examining the length of the bars representing the number of subjects for which the feature was relevant, along with the color of the bars corresponding to the model, one can determine per model which features are the most relevant for each foot condition. It is important to note that while this table provides an overall perspective, it is also possible to identify the most relevant features for each individual patient. This information could be valuable in the diagnosis and treatment for each patient.

**Table 5:** The most relevant five classifier features for each foot condition.

**Tibiotalar osteoarthritis + partial ankle replacement**	
Timing of Min of First Gradient of Tibiotalar flexion (Stance)	
Max of Medial arch angle (Stance)	
Max of Medial arch angle (Swing)	
Min of Medial arch angle (Swing)	
Min of Medial arch angle (Stance)	
**Planovalgus**	
Timing of Max of Inter MT I-V angle (Swing)	
Timing of Min of Inter MT I-V angle (Swing)	
Timing of Min of First Gradient of Medial arch inclination (Swing)	
Timing of Min of First Gradient of Tibiotalar flexion (Swing)	
Min of Medial arch angle (Stance)	
**Consolidated calcaneal fracture**	
Timing of Max of Difference from Normative of Hallux flexion (Swing)	
Timing of Max of Difference from Normative of Subtalar inversion (Stance)	
Timing of Min of Forefoot/ankle supination (Swing)	
Timing of Max of Forefoot/ankle supination (Swing)	
Timing of Min of First Gradient of Tibiotalar flexion (Stance)	
**Hallux rigidus**	
Range of Motion of Tibiotalar flexion	
Range of Motion of Medial arch angle	
Range of Motion of Subtalar inversion	
Timing of Min of First Gradient of Tibiotalar flexion (Swing)	
Timing of Min of First Gradient of Tibiotalar flexion (Stance)	
**Clubfoot**	
Timing of Min of Hallux flexion (Swing)	
Max of Forefoot/ankle abduction (Swing)	
Min of Forefoot/ankle supination (Swing)	
Max of Forefoot/ankle abduction (Stance)	
Min of Forefoot/ankle abduction (Swing)	
**Cavovarus**	
Timing of Min of First Gradient of Medial arch angle (Stance)	
Timing of Max of Inter MT I-V angle |STD| (Swing)	
Timing of Max of Difference from Normative of Medial arch angle (Stance)	
Timing of Min of First Gradient of Medial arch inclination (Swing)	
Timing of Min of First Gradient of Tibiotalar flexion (Stance)	

Colors representing the models: Green, dark blue, orange, light blue, and yellow for RF, KNN, LREG, SVM, and MV, respectively. The bar length is related to the number of subjects for which the feature was relevant.

Reading example of Table 5: In the classification of Tibiotalar osteoarthritis + partial ankle replacement, minimum medial arch angle during both stance and swing phases (the longest bars in the table) were found as the most relevant feature by RF, KNN, and MV models. The other relevant features are to be seen in the table. In the similar manner, the classification of Cavovarus, the timing of minimum first gradient of tibiotalar flexion in stance phase was found the most relevant feature by LREG, SVM, and MV models.

## Discussion

In this study, it is aimed to automate the feature selection and foot condition identification to facilitate the diagnosis of foot conditions and hence, to support experts and physicians in their clinical assessment. An ML pipeline is achieved using the state-of-the-art feature elimination and ML algorithms. LIME is utilized to interpret the outcomes of the ML models so that the experts using this pipeline have feedback about how ML models predict the foot conditions.

Our findings indicate that all the algorithms showed good prediction with a minimum of 0.82 F-1 scores. The success scores of the algorithms are similar but KNN and MV yield the highest scores. When the dataset has a class imbalance, namely some classes have very low data compared to the others, the ML models in general could tend to learn more about the classes with high data. RF is known to overcome class imbalance inherently, however, the test scores in Table 4 indicate that other models also perform well to deal with the class imbalance.

High success scores indicate mathematically strong relationship between the selected features and foot conditions. Moreover, different mathematical features can be computed manually and easily added to the created models. Additionally, automatic feature generation or even featureless classification methods can be explored under the condition that the generated features align with the kinematic characteristics of the joints to ensure interpretability.

Note that the data size is comparably small (i.e. 348 subject data). The models will improve, as the new data arrives or new features are added. Since MV utilizes the other ML models, it will likely have the highest score when there is new data. Another limitation of the data is that averaging across strides may induce non-gait features, especially if the variability among the strides is significant. This variability is reduced by asking participants to walk in their self-walking speed for a certain time before starting the measurement on a walkway to reach a fairly stable walking condition. It was shown by some studies that the gait cycle becomes significantly reliable after certain walking strides^53,54^. Upon achieving a stable walking condition, stride variability becomes a valuable measure, as it can signify the underlying foot condition. Consequently, the study includes standard deviation values to capture this aspect. Furthermore, this type of averaging has been performed with patients who have foot disorders, ambulation, or amputation^26,55–58^. However, analyzing stride data directly; i.e. without averaging, could provide deeper insights across strides and enhance the feature set. This approach expands the dataset size by five to seven times. For these reasons, non-averaged data will be evaluated in future studies.

Furthermore, the model can be extended to classify other functional foot conditions that are not included in this study. Progress in digital patient records of specialized treatment centers will facilitate and strengthen the assistance opportunities of ML and XAI in orthopedic diagnostic decision-making. The trained models do not require high computational resources for making the classification. Therefore, they can be easily integrated into any type of software (e.g. web-based) in the future.

Another potential improvement involves leveraging deep learning to enhance model performances, while preserving interpretability. To achieve this, rather than relying solely on time series data, the focus should still be on crafting models that utilize manually extracted features as inputs. This is essential for maintaining relevance in the XAI outcomes regarding the clinicians’ understanding. Although deep learning methods typically need a larger dataset than traditional ML methods and neural networks, few-shot learning techniques could be used to train a model with a limited amount of data. Exploring this approach will be a focus of our future research. The ML methods generate black-box models, meaning that how these models make a prediction is not transparent and very difficult to interpret. It is a common problem in neural network-based models, as well and there are new methods to interpret the resulting predictions of the models. In this study, LIME was employed for the interpretation of the model predictions by showing the most relevant features in the prediction. Furthermore, if the same features are selected by most of the models, it can be stated that those features might have strong relevance in the classification. Through computational analysis, Wang et. al. conducted a study on the biomechanical effects of total ankle replacement (total ankle arthroplasty) and ankle arthrodesis during the stance phase, specifically on the inner foot. Their findings revealed that the deviations in ankle join motion are compensated by angular displacement in the fore-foot, resulting in different force transmission among segments, joint contact pressure, and bone stress distribution compared to intact foot^59^. Tibiotalar osteoarthritis + partial ankle replacement might have similar effects. Considering the LIME results, it can be said that these effects exert a significant influence on the medial arch in both stance and swing phases. In cases of Planovalgus and Cavovarus, the majority of relevant features are associated with medial arch and tibiotalar flexion. This alignment with these foot conditions is logical since compared to typical feet, Planovalgus and Cavovarus are characterized by lower and higher medial arch, respectively. For Clubfoot, the most relevant features pertain to foot abduction and supination, which is again logical since in this foot condition, the foot is turned inward.

Upon evaluating the LIME results across different algorithms, it becomes evident that particular algorithms work better to identify relevant features associated with specific foot condition. Remarkably, in cases of Planovalgus and Clubfoot, certain features are exclusively found as relevant by an individual algorithm (RF, KNN, LREG, or SVM). For instance, in the case of Clubfoot, min of forefoot/ankle abduction in swing phase was identified only by RF and MV. The employment of MV allows us to harness the distinctive strengths of all the algorithms within a unified MV model. This is reflected in the LIME results, where MV emerges as the most effective method for identifying nearly all of the relevant features. It is possible that there could be a relation between operation of the ML model that exclusively identified relevant features across the patients and the gait characteristics of the foot for a specific foot condition as well as the degree of dispersion of features among patients. This point will be considered in future studies.

Our study is pioneering the generation of new insights for clinicians, potentially paving the way for future research driven solely by clinical priorities and concerns. Note that for a comprehensive understanding of the clinical relevance of these findings, the LIME results will be discussed with the experts and physicians for the validation of the gained insights. The LIME results may help them discover unknown features that might be critical in a successful inspection of the foot conditions.

## Conclusions

We built an ML pipeline including feature selection, classification, and an interpretability steps. In the feature selection, we extracted features manually and eliminate the ones with low variance. We compared the ML models and showed their ability in the classification of the foot conditions that might reduce clinicians’ effort on time-consuming evaluation of the gait data. The findings of the explainable AI method (i.e. LIME) indicate that it can help to understand the reason behind the classification results, which might give insight in mining new knowledge.

## Data Availability

We have the approval to use these data retrospectively for this specific study. We may not give these data to any third party without formulating a specific scientific goal (like we did here in our collaboration). Also they are not available upon simple request to the corresponding author but only via a regular study.

## Abbreviations

A-GAS: Automated Gait Assessment Score
AI: Artificial Intelligence
CP: Cerebral Palsy
HFMM: Heidelberg Foot Measurement Method
KNN: K-nearest Neighbor
LDA: Linear Discriminant Analysis
LIME: Local Interpretable Model-agnostic Explanation
LREG: Logistic Regression
LRP: Layer-Wise Relevance Propagation
LSTM: Long Short-Term Memory
ML: Machine Learning
MV: Majority Voting
PCA: Principal Component Analysis
RF: Random Forest
SHAP: Shapley Additive Explanations
Std: Standard Deviation
SVM: Support Vector Machine
XAI: Explainable Artificial Intelligence
